# Waterborne microbial risk assessment : a population-based dose-response function for *Giardia spp*. (E.MI.R.A study)

**DOI:** 10.1186/1471-2458-6-122

**Published:** 2006-05-03

**Authors:** D Zmirou-Navier, L Gofti-Laroche, Ph Hartemann

**Affiliations:** 1 INSERM-ERI 11, Nancy University Medical School – 9 av de la Forêt de Haye, BP 184 – 54505 Vandoeuvre-les-Nancy Cedex, France; 2 Grenoble University Hospital, 38700 La Tronche, France

## Abstract

**Background:**

Dose-response parameters based on clinical challenges are frequently used to assess the health impact of protozoa in drinking water. We compare the risk estimates associated with *Giardia *in drinking water derived from the dose-response parameter published in the literature and the incidence of acute digestive conditions (ADC) measured in the framework of an epidemiological study in a general population.

**Methods:**

The study combined a daily follow-up of digestive morbidity among a panel of 544 volunteers and a microbiological surveillance of tap water. The relationship between incidence of ADC and concentrations of *Giardia *cysts was modeled with Generalized Estimating Equations, adjusting on community, age, tap water intake, presence of bacterial indicators, and genetic markers of viruses. The quantitative estimate of *Giardia *dose was the product of the declared amount of drinking water intake (in L) by the logarithm of cysts concentrations.

**Results:**

The Odds Ratio for one unit of dose [OR = 1.76 (95% CI: 1.21, 2.55)] showed a very good consistency with the risk assessment estimate computed after the literature dose-response, provided application of a 20 % abatement factor to the cysts counts that were measured in the epidemiological study. Doing so, a daily water intake of 2 L and a Giardia concentration of 10 cysts/100 L, would yield an estimated relative excess risk of 12 % according to the Rendtorff model, against 11 % when multiplying the baseline rate of ADC by the corresponding OR. This abatement parameter encompasses uncertainties associated with germ viability, infectivity and virulence in natural settings.

**Conclusion:**

The dose-response function for waterborne Giardia risk derived from clinical experiments is consistent with epidemiological data. However, much remains to be learned about key characteristics that may heavily influence quantitative risk assessment results.

## Background

Infectious organisms resistant to disinfectants, such as protozoa and viruses have caused several outbreaks around the world, including in developed countries. The most noticeable pathogen is *Cryptosporidium *which caused the Milwaukee outbreak [1,2], and several episodes in the US, the UK and Canada [3-6]; but *Giardia lamblia*, the most common intestinal protozoan in the US, is also frequently reported in association with waterborne diseases [7-9]. Cysts may be found in water as a result of fecal contamination from both man and animal. Thus, protozoa pose major challenges to design and maintenance of safe water supplies. Microbial risk assessment is an important tool to manage these risks because it allows water quality standards and other management decisions to be based on quantitative estimates [6,10,11]. The US-EPA, for instance, has recommended that a treatment be provided to ensure that populations are not subject to a yearly risk of infection greater than 10^-4 ^[7,12,13].

The quantitative microbiological risk assessment approach is now well established [9,14-16]. Dose-response functions have been proposed and reviewed for waterborne pathogenic micro-organisms and for microbial food safety [17-23]. The dose-response functions that are currently used in risk assessment studies for protozoa stem from clinical trials where adult healthy volunteers have been exposed to known amounts of well characterized micro-organisms and followed to ascertain the development of infection (faecal excretion, seroconversion). Rendtorff (1954) [24] and Dupont *et al *(1995) [25] performed controlled human feeding trials respectively for *Giardia lamblia *and *Cryptosporidium parvum *(oo)cysts. The exponential and the beta-Poisson model are commonly used to describe these relationships [9,15,17,22]. They are based on the assumptions of independent, single hit action, and random distribution of the micro-organisms in the inoculum. But it must be kept in mind that infection was measured by cyst excretion, not by symptoms. This experimental approach is faced with severe ethical restrictions that limit trials to healthy adult volunteers. Also, issues regarding strain selection make generalization of such data questionable [9]. Hence, variability related to infectivity of different strains and to the immune response of hosts could not be accommodated. These limitations must be taken into account in risk assessment studies [7].

Epidemiological data offer the advantage of being based on natural infection events, to bear on a variety of host populations and on microorganisms that are found in real life situations [9]. Epidemiological studies have already been used to assess dose-response functions in the context of outbreaks, but serious difficulties are encountered for retrospective exposure assessment. For instance, a risk assessment was conducted on the basis of disease risk parameter estimated after the dose-response model developed by Dupont on healthy human volunteers [25], and compared to the observed attack rates during the Milwaukee *Cryptosporidium *outbreak [1] to derive concentration estimates of the pathogen in drinking water; the results proved consistent with the levels that were found in ice samples (1.4 oocysts/L versus 0.79 oocysts/L) [18,26]. Similar conclusions were drawn in another outbreak in Bradford (UK) [15].

The present work uses epidemiological data collected in a non epidemic setting and compares the risk estimates derived from the published dose-response parameters for *Giardia *to the observed incidence data in a general population exposed to environmental strains of micro-organisms.

## Methods

The E.MI.R.A (Epidemiology and MIcrobial Risk Assessment) study was carried out between October 1998 and June 1999, in south-east France. It combined a daily epidemiological follow-up of digestive morbidity among a panel of volunteers, and a microbiological surveillance of drinking water. The E.MI.R.A study design has been described in detail elsewhere [27-29].

### Health survey

Briefly, volunteers have been recruited among communities supplied by 4 public water systems (groups 1 to 4), specifically chosen for their raw water vulnerability : one "pristine" groundwater located in a quarstic environment (1); two vulnerable ground watersheds : a quarstic watershed (2), and an unprotected watershed exposed to livestock and community sewage (3); and one surface water : a lake surrounded by human activities (4). Except in the first group whose water was untreated, water was disinfected by chlorine only, hence allowing for presence of chlorine-resistant pathogens despite absence of indicator bacteria. Each participating family completed a self-administrated daily questionnaire whereby all health problems were to be registered. Morbidity data were retrieved by telephone calls, following a daily 1/5^th ^sampling procedure (each working day of the week one fifth of the families were called, thus allowing complete population surveillance on a weekly frequency). This surveillance scheme allowed a continuous description of digestive morbidity incidence, along with outbreaks detection. An "alert" threshold was based on a pilot study data, and was defined as the occurrence of two incident cases of acute digestive conditions in the same community (but not in the same household) during the same 48 hours. To be considered as distinct, a lag greater than 48 hours was required between 2 episodes. A case of *acute digestive conditions *(ADC) was defined as an episode of abdominal pain, nausea, vomiting and/or diarrhea; a case of *diarrheic episode *(DE) was an episode of diarrhea with at least another digestive condition or fever; and a case of *gastro-enteritis *(GE) was an episode of diarrhea with at least another objective sign (fever or vomiting).

E.MI.R.A volunteers were also asked about their drinking water consumption. Data were collected during a week day and a week-end day by self-questionnaires that explored tap and bottled water intake, type of usage of tap water (cold/hot, with or without additional product) and the place of water consumption (home, school, work).

### Water quality survey

One tap water sample was collected monthly in each study group. Additional tap water samples were also collected in case the alert threshold was exceeded. *Giardia *and *Cryptosporidium *were analyzed after filtration of 100 L of water through a Gelman^® ^Envirochek cartridge. The analytical process used in this study is a modified protocol from the method recommended by the US-EPA [30]; clarification was done by ImmunoMagnetic Separation (IMS) and (oo)cysts enumeration by laser-scanning cytometry (Chemscan). The detection limit was 10 cysts/100 L and the recovery rate averaged 50 percent [31]. Virological quality of water was assessed using RT-PCR for detection of enterovirus, rotavirus and astrovirus [32]. Bacterial analyses were also done to control compliance with drinking water quality regulations (absence of thermotolerant -"faecal"- coliform in 100 ml, of faecal streptococci in 100 ml, and of spores of sulfite-reducing clostridia in 20 ml).

### Statistical analysis

Due to the longitudinal design of the study, with repetitive measurements on the same subjects, the relationship between incidence of digestive morbidity and concentrations of protozoa was modeled using Generalized Estimating Equations (GEE), using a logistic link function. Exposure was accommodated as a quantitative variable where "dose" of ingested pathogens was the product of declared individual tap water intake and *Giardia *concentrations (after Ln transformation). To improve exposure ascription, the relationship was tested for different time lags : more or less than 14 days around the corresponding water sample, more or less seven days, and three days; results of the latter exhibited greater associations and are presented hereafter, meaning that only person-times of observation (36% of the data) and cases (32%) that had occurred within 3 days prior to or after the water samples were considered for this analysis. Incidence of ADC rather than DE or GE was chosen as the health endpoint because, while less specific, it is a more sensitive measure of acute digestive morbidity.

The dose-response function that was derived from our epidemiological data was compared to estimates of infectious risks computed after the dose-response curve established by Rendtorff on healthy human volunteers [24]. The latter is an exponential dose-response curve : Pi = 1 - exp (- r D), where Pi is the individual daily probability of infection, r is an organism-specific infectivity parameter (host-microorganism interaction), and D is the daily ingested dose of parasites. For *Giardia*, r = 0.02 (95 % confidence interval : 0.0098–0.036) [10]. The dose is computed as D = CV, where C is the concentration by liter of cysts in tap water (Log transformed), and V is the individual's consumption of un-boiled cold water per day. This model assumes that : (i) pathogens are randomly distributed at the exposure site; (ii) a fraction of the micro-organisms ingested survive to cause infection; (iii) the survival of an individual organism is independent of the presence of any other organism [33].

Illness is conditional on infection, and the probability of becoming ill can be written as P(ill/dose) = P(ill/inf) * P(inf/dose) [33], where P(inf/dose) is the probability of infection supplied by the Rendorff model, and where P(ill/inf) is the infectivity rate of the germ. Now, risk, ie P(ill/dose), can be directly estimated from our epidemiological data : Risk = I_o _× RR_i_, where RR_i _is the relative risk associated with "dose" i (water intake × Ln [concentration i]), and I_o _is the baseline incidence rate, i.e. when concentration is zero. (Because the logistic link function was used, RR was computed as an odds ratio OR_i_.) Thus, risks attributable to increasing counts of *Giardia *can be compared across the two estimation procedures. Both risk estimates are not exactly identical, however, because not all detected cysts in our study are viable and infectious, and because not all infected subjects suffer acute digestive conditions. For these reasons, direct comparison of the two risk estimates might be misleading. Consequently, an abatement factor was applied to the cysts counts used with the Rendorff model risk estimate to take these differences into account. Three hypothetical values of this abatement factor are presented: 1/1 (meaning that all detected cysts are infectious and cause digestive conditions), 1/2 (ie half detected cysts are expected to be infectious and to cause digestive conditions), and 1/5 (in other words, 80 % of detected cysts are assumed non infectious or associated with asymptomatic infections). The comparative analysis was undertaken using Monte Carlo simulation with 5000 iterations and Latin Hypercube Sampling, using @Risk 3.5 (Palisade corporation). The Rendorff exponential model r parameter was given a triangle distribution (most likely r value = 0.0198, min = 0.0098, max = 0.036) [10]. The OR distribution values were derived from our study results. Results are expressed in relative excess risks (in %), where the baseline incidence rate I_o _is that observed when the Giardia counts were 0/100 L.

## Results

The epidemiological follow-up involved 544 volunteers distributed across the 4 water groups, with 27.5 percent of children (0–14 years old) and 12.1 percent subjects over 60 years old (Table 1). Only four "epidemic alerts" were declared according to our definition, one true outbreak being confirmed (group 3 in February 1999). Throughout the study, the ADC incidence rate was 2.8 cases per person-year (95 % CI : 2.6–3.0), ranging from 2.0 cases per person-year [1.6–2.5] to 4.7 [4.0–5.3] according to the study group. The incidence rate of gastro-enteritis was 0.2 cases per person-year. The point estimate of the baseline incident rate of ADC in absence of *Giardia *was 2.4 cases per person-year. Detailed results on the study population and the observed digestive morbidity are presented elsewhere [27-29].

Total tap water intake follows a normal distribution and varies according to season (average in winter = 1.6 L/d, SD = 0.7 L/d; average in spring = 1.8 L/d, SD = 0.8 L/d). For Monte Carlo simulation, we used our data set on un-boiled tap water intake (0.8 L/d, 0.3 L/d). Detailed description of daily drinking water consumption of E.MI.R.A volunteers has been published [32].

Thirty two tap water samples were analyzed, provided by the eight monthly routine sampling runs in each community between November 1998 and June 1999. Four additional tap water samples were analyzed because of four "epidemic alerts". Throughout the study, 30.6 percent (11 out of 36) of the samples were positive for at least one protozoan : eight for *Giardia*, and three for *Cryptosporidium*. The maximum concentration recorded was 110 cysts/100 L for *Giardia *and 4 oocysts/100 L for *Cryptosporidium*. *Giardia *was found in tap water more often and with higher levels than *Cryptosporidium*. Ten protozoa positive samples out of 11 complied with bacterial criteria (table 2).

The odds ratio (OR) associated with one unit increase of the *Giardia *"dose" is 1.76 (95 % CI : 1.21–2.55) after adjustment on the following covariates : community, age, compliance with bacterial criteria (dummy variable), presence or absence of viral markers for enterovirus and astrovirus (dummy variables) (table 3). Home characteristics, gender, and presence of rotavirus or of *Cryptosporidium *(unfortunately, *Cryptosporidium *data were sparse) showed no association with incidence of ADC. Enterovirus exhibited a paradoxical 'protective' effect. This viral family causes more neurologic than digestive conditions [34; 35] and this statistical artefact is possibly due to enterovirus being mostly present, during this study, when more effective enteric pathogens (*Giardia*, astrovirus [29]) were absent, and conversely.

**Figure 1:**
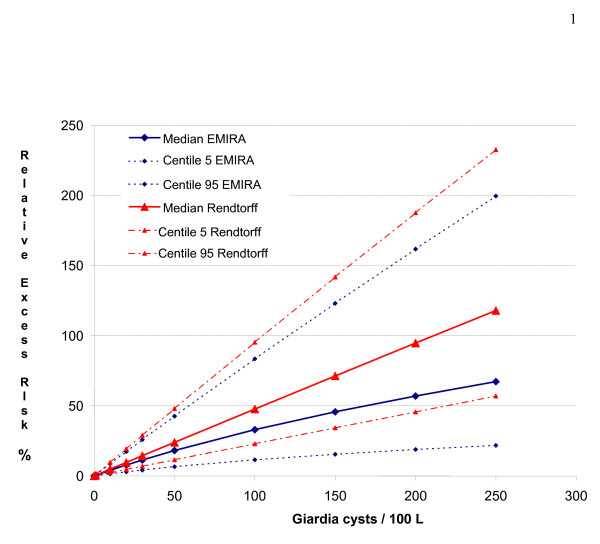
Relative excess risk (in %, and 95% confidence intervals) attributable to Giardia counts in tap water : comparison of results derived from the E.MI.R.A observations (in blue) and from the experimental exponential model (in red) after application of a 20 % abatement factor

When comparing the risk estimates derived from the Rendtorff model and from our observations, the best correspondence is found with a 1/5 abatement factor : for hypothetical Giardia cysts counts ranging between 1 to 200/L (i.e. in the range of measured concentrations), modeled risk values exceed the estimated incidence rate by a factor spanning from 6.5 to 3.0 for a 1/1 abatement factor, and from 3.3 to 1.5 for a 1/2 abatement factor; this ratio did not exceed 1.6 for a 1/5 abatement factor, with values close to 1 for low Giardia concentrations. Assuming a daily water intake of 2 L and a *Giardia *concentration of 10 cysts/100 L, the estimated relative excess risk would be 12 % according to the Rendtorff model (with the 1/5 abatement factor), against 11 % applying the corresponding OR to the baseline rate of ADC in absence of *Giardia *cysts. Figure 1 exhibits risk estimates (expressed in percent increase above the baseline incidence rate, with the 95 % CI limits) from our study results and from the Rendtdorff model values after we applied a 1/5 abatement factor to the cysts counts; given this correction, it shows a good agreement between the two slopes for contamination levels in the domain of observed data (up to 110/L). The underlying assumption in figure 1 is that 20 percent of the detected cysts are capable to induce disease.

## Discussion

The hazard that *Giardia *and *Cryptosporidium *pose to communities supplied by surface water has been early recognized by the US legislation, which requires a minimum of three log removal/inactivation of *Giardia *and two log removal of *Cryptosporidium *[36,37]. These objectives were derived in order to ensure that consumers would not be exposed to a risk greater than one per 10 000 individuals per year [11,19]. In the UK, the "Drinking Water Inspectorate" also takes into account *Cryptosporidium *risks, requiring a maximum level of 10 oocysts/100 L. UK regulations also require that water providers perform a risk assessment on their water treatment facilities and implement continuous monitoring for *Cryptosporidium *[38]. In continental Europe, no rules exist to date for *Giardia *or *Cryptosporidium *in drinking water [39].

While risk assessment is an important tool for management of waterborne infectious risks, it is affected by several uncertainties : there are knowledge gaps about pathogens concentrations, and weaknesses in specification of dose-response functions, amongst other factors [8,9,40]. Dose-response data for infection with *Giardia lamblia *were derived from an experiment on healthy adult [24]. Now, healthy adults are not the most important group from a public health perspective [8,41]. The E.MI.R.A study included some sensitive subpopulations (i.e., children, elderly subjects, pregnant women, but not subjects with chronic diseases), which are excluded from experiments for evident ethical reasons. Gerba *et al *assessed that this subgroups represent about 20 percent of the US population [42].

Our survey provided directly drinking water data; so we did not need to formulate additional hypotheses on pathogens survival at early steps of the water supply chain, a critical uncertainty factor in the waterborne microbial risk assessment procedure [8,40]. The detection method we used, however, does not differentiate between live, virulent and non infectious micro-organisms; nor is it strictly specific of human strains of parasites. On the other side, the imperfect recovery of cysts from water samples may allow for some cysts counts underestimation. The recovery rate of 50 % of the technique we used had not been assessed in a different framework than that of the present study [31], but it stands in the range of values that were published using the same U.S. Environmental Protection Agency method 1623, in the low side [43]. Given these different factors, only a fraction of the detected parasites are viable, infectious and virulent to humans. Several measures of viability have been proposed in the literature, such as germ morphology, *in vitro *excystation assays, infectivity tests in animal or cellular models [8,44,45]. No assay on viability/infectivity was conducted within the E.MI.R.A framework. Teunis *et al *reported that *Giardia *cysts shedding might occur at very low infectious doses (3–10 cysts) [8]. In our study, *Giardia *cysts were found at non negligible levels in tap water (10, 20, 30 and 110 cysts per 100 L).

Other critical issues in exposure assessment should also be discussed. We hypothesised, in this analysis, that one water sample represented the average parasitical quality of water during 7 days (day of sampling and 3 days about). Backing this assumption, a Canadian study showed small differences in *Giardia *cysts counts along consecutive days [46]. It was conducted in highly polluted surface water, however, and whether this observation applies in the low contamination level of the present study is unclear. While we are not aware of published papers describing short term variability of (oo)cysts concentrations in ground or surface water used for drinking water, seasonality has been assessed in several studies, with uneven findings according to watershed vulnerability and to the time pattern of contaminants discharge into surface water [47-51]. Hence, the time representativity of our water samples is not fully characterised despite our repetitive sampling. The latency for *Giardia *infection and disease is commonly reported to be comprised between one and 14 days. Thus, the seven days morbidity period we chose might be incomplete; it showed the best statistical associations, however, compared to 2 weeks or one month sampling frames. Exposure to waterborne pathogens depends upon infectious micro-organisms concentration, but also on the amount of water that is ingested. Most risk assessment studies use 2 L as the default value for daily drinking water intake. Here, we applied the consumption values that were declared by the study volunteers for un-boiled tap water. Finally, one cannot rule out some degree of confounding of the effect attributed to *Giardia *due to concomitant presence of other pathogens, a situation that occurred during the true outbreak that was observed during the study. It should be noted that our risk estimates for *Giardia *are adjusted for other measured concomitant pathogens. In a recent report of the large Bergen waterborne giardiasis outbreak, Norway, the authors underline how difficult it may be to assign cases to a given pathogen, even using sophisticated *Giardia *and *Cryptosporidium *genotyping, both of cases stools and water samples [52,53]. The two protozoans were not found concomitantly in our study, however, but this might be partly explained by imperfect recoveries.

It is reasonable to think that the association between digestive morbidity and *Giardia *exposure differs according to age. Unfortunately, we could not assess this heterogeneity, because of lack of statistical power, the reason why we split our data in only three age categories. The literature does not provide such information either and this calls for further research. Our risk estimates for *Giardia *are consistent with those derived after the Rendtorff model provided that we down-weight the measured germ counts by a 1/5 factor. This parameter encompasses several dimensions: (i) the recovery rate of the analytical techniques is not 100 percent (with the Gelman^® ^protocol used here this rate averages 50 percent); (ii) some detected cysts might be non viable and non infectious to humans; (iii) some infected subjects might be asymptomatic. It is difficult, and out of the scope of this study, to devise what role these different factors play, in the net estimated 20 percent weigh. For instance, one could hypothesise that the incidence rate could have been greater in this general population, because the viability and infectivity characteristics of the pathogens in the natural environment might be larger than those assayed in the Rentdorff experience. In their 1991 work, Le Chevallier *et al *had found that 13.3 percent of the detected cysts had a viable morphology [44]. A probability of disease, when infected, of about 70 percent (95 % CI : 35–100) has been assessed for *Cryptosporidium *[54] and 37 % of oocysts found in six US watersheds showed viable and infectious [36]. For *Giardia*, symptomatic infection proportions have been reported ranging between 50 % and 67 %, and as high as 91 % [7]. On the other hand, there might be some degree of population immunity, associated with the prevalence of cysts in drinking water [55,56]

While substantial efforts are ongoing to improve risk assessment of *Cryptosporidium*, because of its well established virulence for immunocompromised subjects, fewer risk assessment data concern *Giardia*. The E.MI.R.A study had been designed to deal with the 2 protozoa, but presence of *Cryptosporidium *in tap water of our French communities was too rare an event for analysis. Evidence of consistency between estimated and observed germ counts after the Milwaukee *Cryptosporidium *outbreak also gives some support to the validity of risk parameters that were computed through clinical infection assays; we suggest, however, that studies along the same rationale as the E.MI.R.A. study could be designed to assess dose-response functions for this pathogen in a non-epidemic context, because waterborne exposure to protozoa may result in sporadic cases and not necessarily in outbreak situations, as it was seen in the present study. In an Australian study in Sydney, raw and finished water were contaminated by *Cryptosporidium *and *Giardia *without discernable increases in illness in the general population [57]. It is well known, however, that only a very small fraction of individuals with acute digestive conditions ask for medical attention and are adequately diagnosed and registered [5].

## Conclusion

Quantitative microbial risk assessment is a useful tool for the management of waterborne infectious risks. This study, conducted in a general population in a natural setting, suggests that the exponential model for waterborne *Giardia *risk derived from clinical experiments is consistent with epidemiological data. However, much remains to be learned about key characteristics that may heavily influence quantitative risk assessment results, such as germ viability, infectivity and virulence, or analytical recovery performances in natural environments.

## Competing interests

The author(s) declare that they have no competing interests

## Authors' contributions

D. Zmirou-Navier designed the study and participated in writing the manuscript. L. Gofti-Laroche conducted the study, performed the statistical analysis and participated in writing the manuscript. Ph Hartemann helped to draft the manuscript. All authors read and approved the final manuscript.

## Pre-publication history

The pre-publication history for this paper can be accessed here:



## Figures and Tables

**Table 1:** Demographics of the population, by study group (n = 538)

	**Group 1 **(n = 116)	**Group 2 **(n = 100)	**Group 3 **(n = 100)	**Group 4 **(n = 222)
**Sex ratio**	1.04	0.79	0.87	1.05

**Age structure (%)**				
< 15 years	27.6	20.0	26.0	31.5
15–59	64.6	59.0	61.0	58.6
>= 60 years	7.8	21.0	13.0	9.9

**Table 2:** Microbiological surveillance of tap water*

	Group 1				Group 2				Group 3				Group 4			
	G	**C**	**V**	**B**	G	**C**	**V**	**B**	G	**C**	**V**	**B**	G	**C**	**V**	**B**
Run 1	**<**	**<**	**<**	**-**	**<**	**<**	**<**	**+**	**<**	**<**	**<**	**-**	**<**	**<**	**+**	**-**
Run 2	**30**	**<**	**<**	**-**	**10**	**<**	**<**	**-**	**<**	**<**	**+**	**-**	**<**	**<**	**+**	**-**
Run 3	**<**	**<**	**<**	**-**	**<**	**<**	**<**	**-**	**<**	**<**	**<**	**-**	**110**	**<**	**<**	**-**
Run 4	**10**	**<**	**<**	**-**	**20**	**<**	**<**	**-**	**10**	**<**	**+**	**-**	**<**	**<**	**+**	**-**
Run 5	**<**	**<**	**<**	**+**	**<**	**<**	**+**	**-**	**<**	**<**	**+**	**-**	**<**	**<**	**+**	**-**
Run 6	**<**	**<**	**<**	**-**	**<**	**<**	**<**	**-**	**<**	**3**	**<**	**+**	**<**	**1**	**+**	**-**
Run 7	**10**	**<**	**<**	**-**	**<**	**<**	**+**	**+**	**<**	**<**	**<**	**-**	**<**	**<**	**+**	**-**
Run 8	**<**	**<**	**<**	**+**	**<**	**4**	**<**	**-**	**<**	**<**	**<**	**-**	**<**	**<**	**<**	**-**
Alert 1	**<**	**<**	**<**	**-**												

Alert 2													**<**	**<**	**<**	**+**
Alert 3									**10**	**<**	**+**	**-**				
Alert 4					**<**	**<**	**<**	**-**								

* < = below detection limit. G = Giardia cysts counts (N per 100 L); C = Cryptosporidium oocysts counts (N per 100 L); V = no detection (<) or detection (+) of viral RNA (enterovirus, rotavirus, or astrovirus); B = compliance (-) or non compliance (+) with bacterial indicators' limit values

**Table 3:** Results of the multivariate analysis

Variable name	*Odds Ratio*	95% CI	P value
**Study group (class)**			
2	0.83	[0.65–1.06]	0.13
3	1.19	[1.01–1.39]	0.03
4	0.84	[0.76–0.93]	0.001
**Age (class)**			
15–59 years-old	0.95	[0.85–1.06]	0.36
> 60 years-old	0.95	[0.82–1.11]	0.54
**Bacterial Compliance**	0.81	[0.64–1.02]	0.08
**Enterovirus RNA (+/-)**	0.61	[0.46–0.80]	0.0003
**Astrovirus RNA (+/-)**	1.49	[1.16–1.90]	0.002
V*Ln (Giardia)*	**1.76**	**[1.21–2.55]**	**0.003**

* combination of tap water intake and *Giardia *counts : V*Ln(*Giardia) *Other non significant variables in the model : Gender, Socio-economic status, Housing, Rotavirus RNA (+/-) and *Cryptosporidium *counts
